# Comparative analysis of microRNA expression profiles between A549, A549/DDP and their respective exosomes

**DOI:** 10.18632/oncotarget.15009

**Published:** 2017-02-02

**Authors:** Xiaobing Qin, Shaorong Yu, Xiaoyue Xu, Bo Shen, Jifeng Feng

**Affiliations:** ^1^ Research Center for Clinical Oncology, Nanjing Medical University Affiliated Cancer Hospital, Jiangsu Cancer Hospital and Jiangsu Institute of Cancer Research, Nanjing, Jiangsu Province, China; ^2^ Department of Oncology, Xuzhou First People's Hospital, Xuzhou, Jiangsu Province, China

**Keywords:** lung cancer, microRNA, exosomes, drug resistance, cisplatin

## Abstract

Exosomes were reported to transport bioactive molecules and influence the biology behavior of recipient cells. In order to study the role of exosomal microRNAs in the mechanism of cisplatin resistance to lung cancer cells, we analyzed the expression profiles of microRNAs in A549, A549/DDP cells and their exosomes by microarray. The results showed that a certain proportion of microRNAs were co-expressed in the cells and exosomes. Linear regression analysis showed that the expression of microRNAs in A549 and A549/DDP cells were strongly correlated with those in their respective exosomes. The expression level of 5 microRNAs (miR-197-5p, miR-4443, miR-642a-3p, miR-27b-3p and miR-100-5p) with the most differential expression were verified by qRT-PCR. The results were consistent with those of the microarray. Target gene prediction and pathway analysis discovered that the microRNAs in the intersections may participate in drug resistance. And the prediction of their association with diseases found that most of these microRNAs was associated with lung cancer. We could draw a preliminary conclusion that microRNAs in exosomes may be involved in the drug resistance of lung cancer cells to cisplatin.

## INTRODUCTION

Lung cancer is one of malignant tumors which can maximally threaten the population's health and life worldwide. Global Cancer Statistics in 2012 revealed that lung cancer was the leading cause of cancer death among males in both more and less developed countries, and has surpassed breast cancer as the leading cause of cancer death among females in more developed countries [1]. Although lung cancer patients can be treated with a series of standard treatments, the five-year survival rate of lung cancer is still not satisfactory. Drug resistance is the main reason for treatment failure and results in poor prognosis.

Recent studies [2, 3] indicated that there are a large number of vesicles of which the diameters are varying from 30nm~100nm in body fluid. These vesicles are encapsulated by the lipid bilayer. They are called as exosomes. The nanometer-level extracellular vesicles can be released by almost all kinds of cells and can confer the intercellular communication of cells under physiological or pathological conditions. Exosomes can be used as the biomarkers of many diseases since it contains protein, DNA, RNA, microRNA(miRNA), lncRNA, and so on. Exosomes are distributed in various human body fluids, including blood plasma/serum [4], breast milk [5], saliva [6], cerebrospinal fluid(CSF)[7], ascite [8], urine [9], and semen [10]. Exosomes are involved in tumor occurrence, development, invasion, metastasis, immunosuppression, drug resistance and can also be applied in clinical diagnosis and treatment [11, 12]. Studies showed that the treatment of tumor can be achieved through exosomes [13]. For example, exosomes can act as drug carrier, so as to achieve the therapeutic effect through carrying miRNA into the target cells and aiming to specific target genes [14].

MiRNA is an endogenous single-stranded non-coding micro RNA molecule. The length is 18~24nt. It can specifically bind with 3′-untranslated region (3′-UTR) in target mRNA, induce its degradation or inhibit its translation, so as to effectively silence the target gene protein expression at the post-transcriptional level [15, 16]. Studies have shown that miRNAs play an important role in the physiological and pathological processes, and are involved in all tumor related processes, such as tumorigenesis, differentiation, proliferation, apoptosis, invasion and metastasis [17, 18].

As a transport tool for miRNAs, exosomes play important roles in the exchange of information between cells [19]. Researchers found miRNAs transported by exosomes are of great value in drug resistance of cancer cells. For example, Shanliang Zhong et al [20] found in breast cancer that a number of miRNAs were concentrated in exosomes and participate in the process of drug resistance. Yu DD et al. [21] demonstrated that exosomes can effectively transmitted drug resistance in breast cancer, and the delivery of miR-222 via exosomes can serve as the mechanism. However, there is little research on the role of exosomal miRNAs in the process of lung cancer cells to cisplatin(DDP) resistance. Therefore, it is particularly valuable to analyze the differential expression of miRNAs in parental cells, their resistant cells and the corresponding exosomes.

Our previous study suggested that lung cancer cells can secrete exosomes to microenvironment after DDP treatment, these exosomes can improve drug resistance of recipient cells to DDP [22]. In order to further study the role of exosomal miRNA in the mechanism of DDP resistance in lung cancer cells, we first established DDP resistant lung cancer A549 cells(A549/DDP). Then the expression profiles of miRNAs in A549 cells, A549/DDP cells and their respective exosomes were analyzed by microarray. To the best of our knowledge, this was the first study reporting the miRNA expression profiles of DDP-resistance lung cancer cells and their exosomes compared with their parental ones, as well as their association with DDP resistance.

## RESULTS

### Establishment of A549/DDP cell line

We treated parental A549 cells with stepwise increasing concentrations of DDP, and the final DDP concentration is 1 μg/ml. In order to assess the resistance index of A549/DDP cells, cell viability was determined by Cell Counting Kit-8 (CCK-8) assay. A549 and A549/DDP cells were treated with different concentration of DDP. Results showed that the inhibitory concentration to produce 50% cell death (IC50) values of DDP were 1.93±0.53 μg/ml and 21.15±1.07 μg/ml for A549 and A549/DDP, respectively. The resistance index is more than 10 folds, which indicates that A549/DDP cells are resistant to DDP.

### Characteristics of exosomes

**Figure 1:**
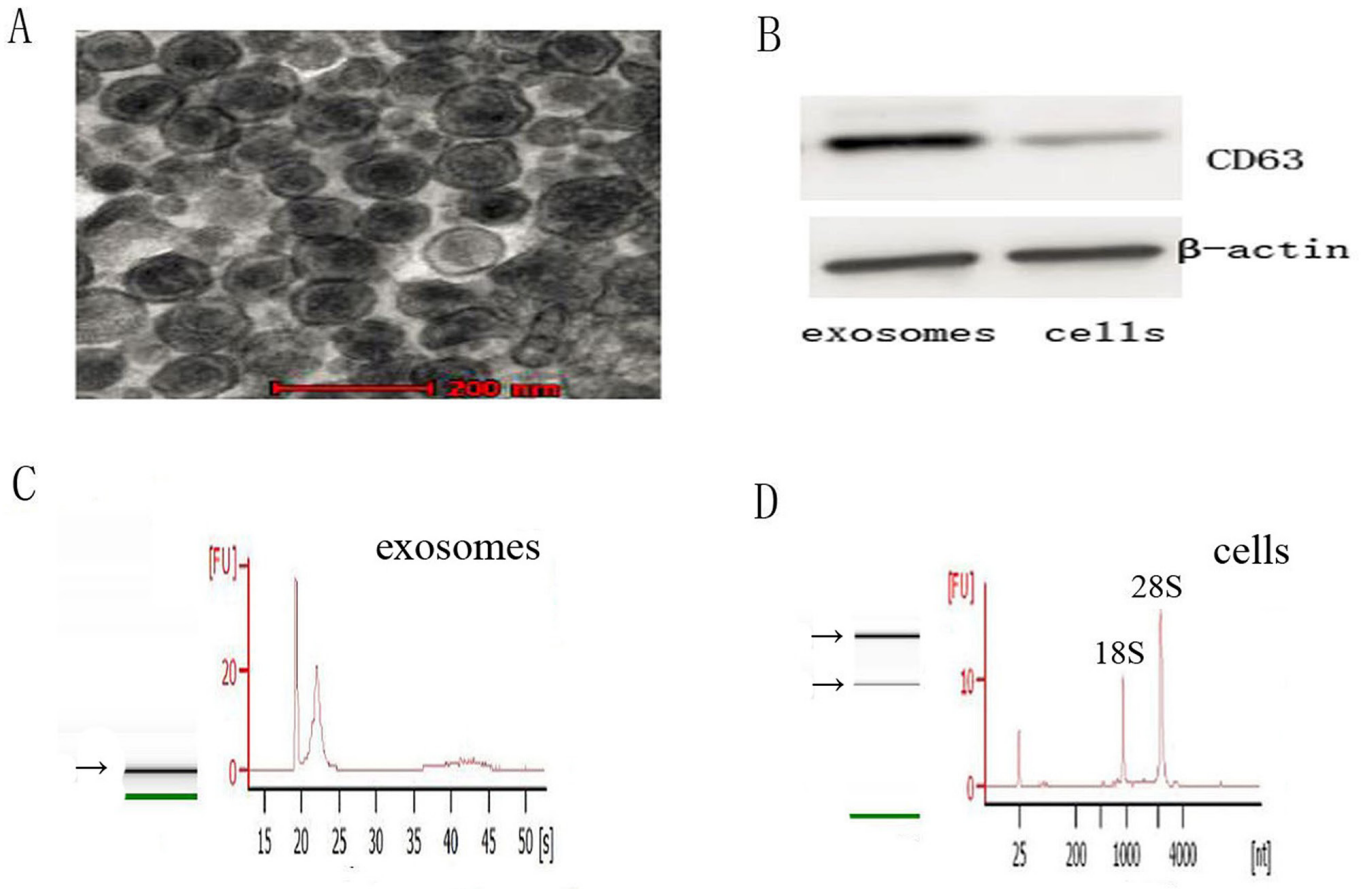
Characteristics of exosomes and the RNA from exosomes and cells **A**. Representative TEM photograph of exosomes (scale bar, 200nm). **B**. Western blot of exosomes and cells for CD63 and β-actin. **C**. and **D**. The analysis of RNA from exosomes and cells shows that exosomes are enriched in small RNAs and contain no 18S and 28S ribosomal RNAs, while cells contain 18S and 28S ribosomal RNAs.

Circular, cup - shaped, double - layer membrane vesicles were observed under the Transmission electron microscope (TEM) (Figure 1A). The diameter of exosomes was during 30-100nm. Western blot showed that CD63 was expressed in exosomes and was not detected in cells (Figure 1B). These results proved that the extracted vesicles were exosomes. Exosomes could be successfully extracted by supercentrifugation.

### The difference characterization of RNA between exosomes and cells

RNA of exosomes and cells were analyzed using a Bioanalyzer Nano assay and capillary electrophoresis. The results revealed that the RNA content were strikingly different between exosomes and cells of A549 and A549/DDP (Figure 1C and 1D). RNA of exosomes is enriched in small RNAs and contain no 18S and 28S ribosomal RNAs, whereas RNA of cells is more than 200 bases and contain 18S and 28S ribosomal RNAs.

### The miRNA expression profiles of A549 cells, A549/DDP cells and their respective exosomes

**Figure 2:**
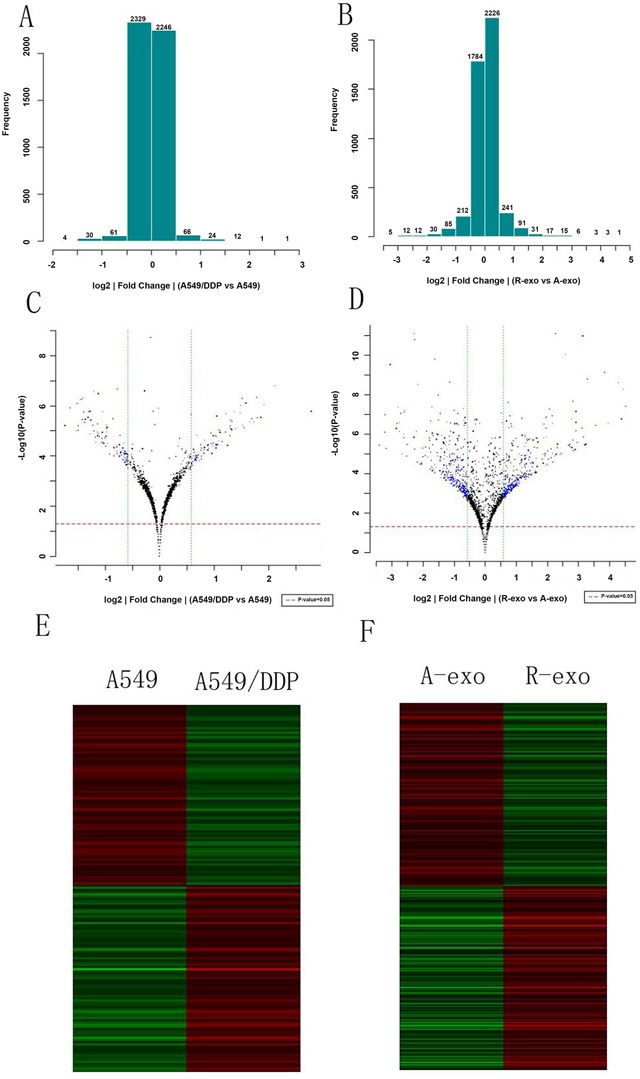
MiRNA expressions profiles of A549, A549/DDP and their exosomes **A**. and **B**. Fold difference histograms. They showed the differential distribution of signals in all detected gene probes of the two cell lines and exosomes. **C**. and **D**. The volcano plots. They showed the distribution of differential gene probes in comparison. **E**. and **F**. Cluster analysis figures. Each row represented one gene probe. The red and green levels indicated the up-regulated/down-regulated amplitude of probe signal. (A-exo represents exosomes of A549, R-exo represents exosomes of A549/DDP).

Fold difference histogram (Figure 2A and 2B) showed the differential distribution of signals in all detected gene probes (control probe and flagged probe excluded). This histogram tended to be normal distribution, suggesting that the up-regulated and down-regulated genes in comparison were roughly equivalent. The volcano plots (Figure 2C and 2D) showed the distribution of differential gene probes in comparison. The red/green dashed lines represented the P value and threshold value of fold screening. Every point was the detected gene probe (excluding control probe and flagged probe). Differential expressed genes were defined when fold change > 1.5 (|log2 ratio| > 0.585) and P value < 0.05. Accordingly, compared with A549 cells, 85 up-regulated genes and 82 down-regulated genes were detected in A549/DDP cells; compared with the exosomes secreted by A549, 346 up-regulated genes and 307 down-regulated genes were detected in A549/DDP exosomes. These differential genes were further analyzed. Figure 2E and 2F were the cluster analysis figures. Each row represented one gene probe. The red and green levels indicated the up-regulated/down-regulated amplitude of probe signal. 167 differentially expressed gene probes in cells and 269 differentially expressed gene probes in exosomes, whose difference ratio between maximum and minimum was more than 100 times, were included in the analysis.

### The intersection of miRNA expression profiles between cells and exosomes

**Figure 3:**
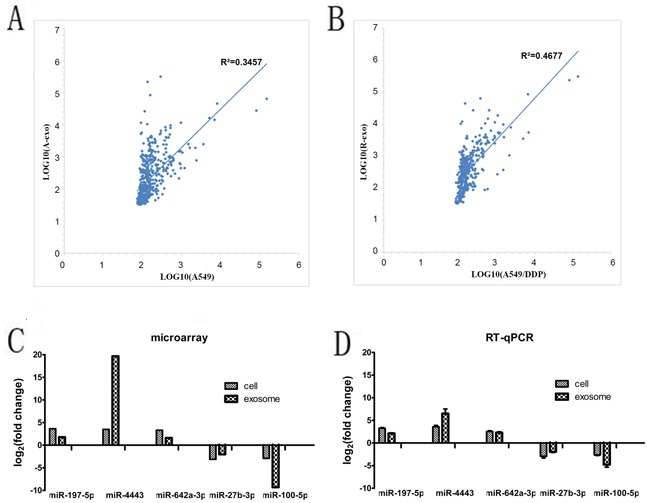
The intersections of miRNAs in the cells and exosomes **A**. and **B**. Linear regression analysis showed that there was significant correlation of miRNA expression profiles between A549 cells and their exosomes, the same situation was found between A549/DDP cells and their exosomes. **C**. and **D**. The expression levels of the most 5 differentially expressed miRNAs in the cells and exosome by microarray and qRT-PCR. (A-exo represents exosomes of A549, R-exo represents exosomes of A549/DDP).

Intersection of miRNAs which is significantly downregulated both in A549/DDP cells compared with A549 cells and in A549/DDP exosomes contrast to A549 exosomes was analyzed. Intersection of significantly upregulated miRNAs was examined as well. 11 up-regulated genes and 31 down-regulated genes were obtained. In all 898 miRNAs which were found in A549 cells and their exosomes, we found that 237 (26.4%) miRNAs were expressed in both cells and exosomes. Similarly, in all 535 miRNAs analyzed in A549/DDP cells and their exosomes, 181 (33.8%) miRNAs were detected both in A549/DDP cells and the exosomes. Linear regression analysis (Figure 3A and 3B) showed that there was significant correlation of miRNA expression profiles between A549 cells and their exosomes, the same phenomenon was found between A549/DDP cells and their exosomes. MiR-197-5p, miR-4443, miR-642a-3p, miR-27b-3p, miR-100-5p, miR-3676-5p, miR-125b-5p, miR-29a-3p, let-7i-5p and miR-3195 were the top ten differentially expressed miRNAs. MiR-4443 was the most significant up-regulated miRNA in A549/DDP exosomes. MiR-100-5p was the most remarkable down-regulated miRNA in A549/DDP exosomes.

### QRT-PCR validation of microarray results

To validate the results of the microarray, we further chose the 5 most differentially expressed miRNAs(miR-197-5p, miR-4443, miR-642a-3p, miR-27b-3p and miR-100-5p) in the cells and exosome for qRT-PCR. Primer sequences are shown in Table 1. The results of qRT-PCR showed that the 5 miRNAs were differentially expressed with the same trend and reached statistical significance (P < 0.05) (Figure 3C and 3D), which indicated that the results of qRT-PCR were consistent with those of microarray.

**Table 1:** Primers used for RT-qPCR

miRNA	Forward primer(5′-3′)	Reverse primer(3′-5′)
miR-197-5p	ACACTCCAGCTGGGTCGGGTAGAGAGGGCAGT	TGGTGTCGTGGAGTCG
miR-4443	ACACTCCAGCTGGGTTTGGAGGCGTG	TGGTGTCGTGGAGTCG
miR-642a-3p	ACACTCCAGCTGGGTAGACACATTTGGAGAG	TGGTGTCGTGGAGTCG
miR-27b-3p	ACACTCCAGCTGGGTTTCACAGTGGCTAAG	TGGTGTCGTGGAGTCG
miR-100-5p	ACACTCCAGCTGGGTAACCCGTAGATCCGAA	TGGTGTCGTGGAGTCG

### Prediction of target gene and pathway analysis

The target gene prediction and enrichment analysis of 11 up-regulated miRNAs as well as 31 down-regulated miRNAs were performed. The target genes of the selected dysregulated miRNAs were predicted using TargetScan [23] (Release 7.0, http://www.targetscan.org) and miRDB [24] (Version 5.0, http://www.mirdb.org). Considering that these procedures had a certain false positive rate, only the genes predicted by two procedures could be considered as the target gene of miRNA. We found that 25,779 target genes were detected of these miRNAs (the target genes were failed to be predicted by miRNA-3676-5p and miRNA-210). One miRNA might regulate hundreds of target genes. The same gene might also be regulated by multiple miRNAs.

**Figure 4:**
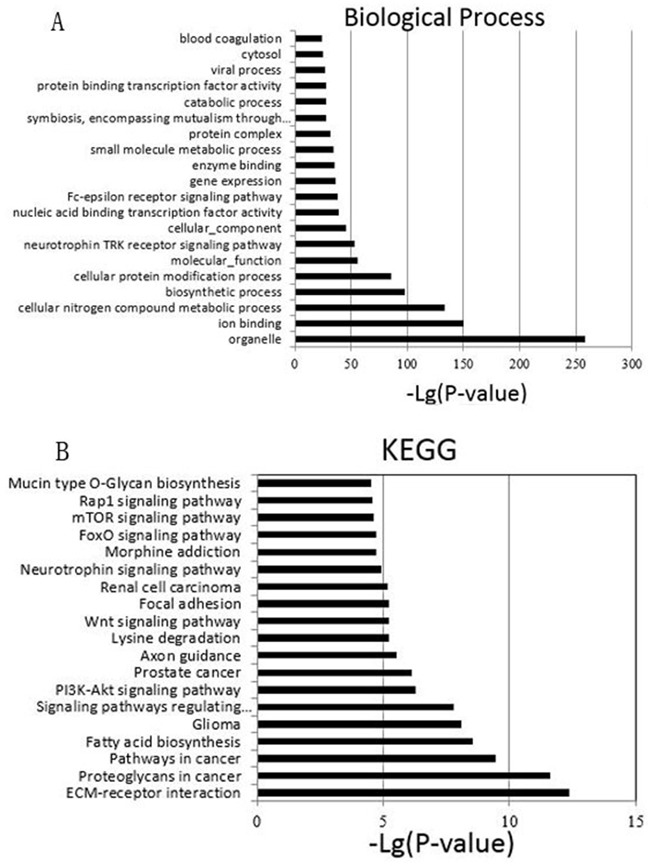
**A**. The top 20 GO terms of predicted targets belong to the differentially expressed miRNAs. **B**. Pathway analysis of differential expression genes by KEGG database. The top 20 are listed in the graph.

Then, we employed a Gene Ontology (GO) approach using Database for Annotation, Visualization and Integrated Discovery [25] (DAVID, Version 6.7, http://david.abcc.ncifcrf.gov), an unbiased prediction program. Among the top 20 significant terms belonging to biological process (Figure 4A), the most GO category was related to organelle. Other GO categories such as ion binding, cellular nitrogen compound metabolic process, and biosynthetic process were also enriched. To characterize the predominant pathways, we next assigned putative targets into Kyoto Encyclopedia of Genes and Genomes (KEGG) analysis. KEGG pathway enrichment analysis was performed to compare the specific miRNAs targets with the whole reference gene background. The count number larger than 2 and P-value less than 0.05 were chosen as cut-off criterion. The top 20 signal pathways were shown in Figure 4B. Among the 20 pathways, PI3K-Akt signaling pathway, Wnt signaling pathway and mTOR signaling pathway have been confirmed to be correlated with multidrug resistance, since treatment failure of lung cancer is partly related to these pathways [26–28]. Our data indicated that these differentially expressed miRNA may be involved in the drug resistance of lung cancer cells.

### Prediction of association of these miRNAs with diseases

**Figure 5:**
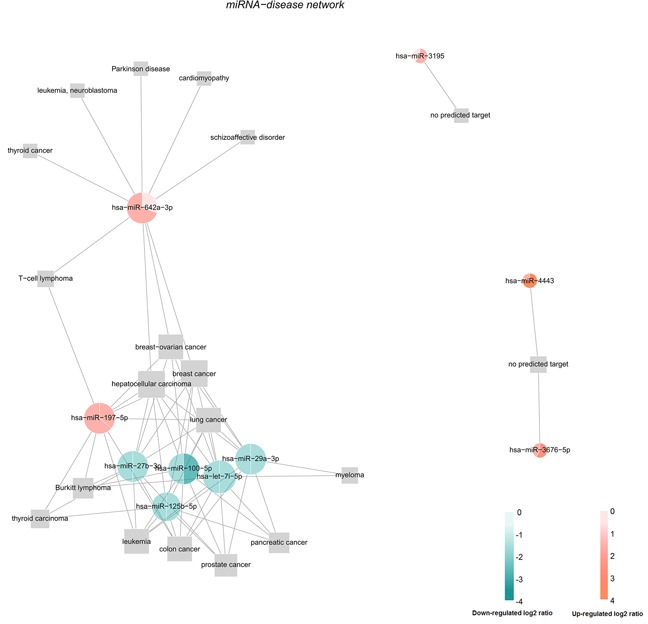
The miRNA-disease network The prediction of the association between the top ten differentially expressed miRNAs with diseases were performed using MDAP.

Identifying the relationship between miRNAs and diseases not only contributes to a better understanding of their potential in clinical, for example new approaches by miRNA delivery may be developed to treat cancer patients. It can also help to understand the genetic network mechanisms [29–31]. The prediction of the association between the top ten differentially expressed miRNAs with diseases were performed using MicroRNA-Disease Association Prediction(MDAP) (http://server.malab.cn/MDAP/). Results show that most of these miRNAs (miR-197-5p, miR-27b-3p, miR-100-5p, miR-125b-5p, miR-29a-3p and let-7i-5p) were correlated with lung cancer, whereas miR-642a-3p has no correlation with lung cancer. There are some miRNA not getting the forecast results, such as miR-4443, miR-3676 and miR-3195. Figure 5 is the miRNA-disease network. The red labeled miRNA was up-regulated and the green marker was down-regulated. Our data suggested that most of these miRNAs may participate in tumorigenesis and development of lung cancer. However, these are only preliminary prediction results. Further experimental studies are needed to investigate whether a miRNA can be applied in clinical.

## DISCUSSION

In the present study, we analyzed the miRNA expression profiles of lung cancer cells and exosomes resistant to DDP compared with the parental ones by microarray. Our results showed that miRNAs were contained in exosomes, and they may be involved in the drug resistance of lung cancer cells to DDP.

DDP is one of the most basic drugs for the treatment of lung cancer. Drug resistance play an important role in the treatment failure of lung cancer patients. Many clinical trials concerning the prediction of DDP efficacy have failed and there is no suitable biomarker yet [29–32]. Why these biomarkers were not suitable in clinical was potentially because they can't reflect the patient's situation dynamically and overall. Therefore a dynamic biomarker to predict patients’ response to DDP is needed and exosome is an ideal choice.

Exosomes are nanometer-sized membranous vesicles involved in cell-to-cell communication. Existing evidences show that tumor-derived exosomes are involved in cancer development, metastasis, drug resistance, and immunity, and they can be used as biomarkers for diagnosis and prognosis [3, 33]. The stable presence of miRNAs in body fluids is partly because they are packed into microvesicles or exosomes [13]. Many studies showed that miRNAs in exosomes can affect the sensitivity of tumor cells to drugs. For example, Keith et al. [34] discovered in triple-negative breast cancer that delivery via miR-134-enriched exosomes can reduce cellular migration and invasion, and enhance sensitivity to anti-Hsp90 drugs. However, there were few studies concerning the mechanism of exosomal miRNA on DDP resistance to lung cancer cells. It can speculated from our research in 2014 [22] that exosomes were involved in the sensitivity regulation of lung cancer cells to DDP. However, which miRNA has altered and what kind of changes have occurred remains unclear. In order to further study whether A549/DDP drug resistant cells could transmit drug resistance to the sensitive cells by exosomal miRNA, A549/DDP drug resistant cells were established, the exosomes were successfully extracted, and the differentially expressed miRNAs in the cells and exosomes were analyzed by microarray.

We examined the intersection of miRNAs between the cells and the exosomes. By companion, it has been found that a certain proportion of miRNAs were co-expressed in the cells and exosomes. Some of the expression of miRNAs in the exosomes were higher than in the cells, and some were lower. So we assumed that some miRNAs in exosomes were shared with their original cells, they may be involved in the alternation of DDP resistance in lung cancer cells, which was consistent with the results of other scholars in other cancers [35–38]. Target gene prediction and GO and KEGG enrichment analysis of these miRNAs showed many miRNAs had specific target genes in PI3K-Akt, Wnt and mTOR signal pathways. These signal pathways were confirmed to be involved in drug resistance and treatment failure. It was found from the prediction of the association between miRNA and disease that most of our differentially expressed miRNAs have potential associations with lung cancer, which are vital for the further investigation. We also compared the differentially expressed miRNAs with other scholars’ researches, their results supported that some of these miRNAs were involved in regulating the sensitivity of cancer cells to chemotherapy [39, 40]. However, further studies are still needed to investigate how these exosomal miRNA play in the process of DDP resistance of lung cancer.

In conclusion, we identified some dysregulated miRNAs in the intersection of genes between the cells and the exosomes of A549 and A549/DDP. MiRNAs in exosomes may be participate in DDP resistance in lung cancer cells. Our research has laid a good foundation for future research concerning the relationship between exosomal miRNA and DDP resistance in lung cancer. Whether the drug resistance can be reversed through these exosomal miRNA and whether the targeted drug delivery can be achieved through drug carrier will also be explored. We believe that the intervention of exosomal miRNA may provide a new strategy for the diagnosis and treatment of lung cancer patients in future.

## MATERIALS AND METHODS

### Cell culture

Human lung adenocarcinoma cell A549 was purchased from Cell Bank of Chinese Academy of Sciences (Shanghai, China). A549/DDP cell line was established from A549 in our laboratory, by exposing A549 to gradually increasing concentrations of DDP, and the final DDP concentration is 1 μg/ml. All cells were grown in RPMI-1640 medium (Nanjing Kaiji Biology, China) containing 10% fetal bovine serum (FBS) (Gibco, USA). Cells were incubated at 37°C and 5% CO_2_ in a humidified atmosphere. In order to avoid the influence of drug to the cells, they were cultured in drug-free medium for at least 2 weeks before subsequent experiments. Parental A549 cells were cultured as a control synchronously, unexposed to DDP.

### Cell counting kit-8 assay (CCK-8)

The IC50 of A549 and A549/DDP was measured by Cell Counting Kit-8 assay. Briefly, the cells were seeded into 96-well plates at a density of 5×10^3^ cells/well and exposed to different concentrations of DDP for 48 h. 10 μL of freshly prepared CCK-8 solutions(Dojindo, Japan) were added into each well and the optical density (OD) was measured at 450 nm using a scanning multi-well spectrophotometer (Bio-Rad Model 550, USA). Each combination of drug concentration was set up in three wells and repeated at least three times.

### Isolation of exosome

In order to avoid the impact of exosomes, the following experiments use exosome-depleted FBS. FBS was depleted of exosomes by ultracentrifugation at 10^6^ g at 4°C for 16 h (Beckman Coulter Avanti J-30I, USA). After being incubated for 48-72 h, the culture medium was harvested and isolated by ultracentrifugation. Briefly, cell culture media was sequentially centrifuged at 300 g for10 min, 2,000 g for 15 min, and 12,000 g for 30 min to remove floating cells and cellular debris. The supernatant was further ultracentrifuged at 100,000 g for 2 h at 4°C, washed with phosphate-buffered saline (PBS), and submitted to a second ultracentrifugation at 100,000 g for 2 h at 4°C. The final exosome pellets were used immediately. Exosomal protein concentration was quantified by BCA protein assay kit (Beyotime Biotechnology, China).

### Transmission electron microscope (TEM)

For electron microscope observation, the exosomes were precipitated and fixed in 2.5% glutaraldehyde at 4°C immediately. After fixation, the specimens were processed through dehydration in gradient alcohol, infiltration in epoxy resin and embedded. The ultrathin sections were stained with uranyl acetate and lead citrate and observed under TEM (JEOL JEM-1010, Japan).

### Western blot

The cells and exosomes were lysed in RIPA buffer (Beyotime Biotechnology, China). The protein samples were separated by SDS-PAGE and transferred to a polyvinylidene fluoride membrane. Rabbit polyclonal CD63 antibody (SBI, USA) was used at a dilution of 1:1000 and the secondary antibody(SBI, USA) at 1:20,000 dilution. β-actin antibody (Cell Signaling Technology, USA) was used at 1:1000 dilution. The bound antibodies were detected using ECL Western Blotting Detection system.

### RNA exaction from exosomes and cells for microarray analysis

Total RNA Purification Kit (Norgen, CA) was used to extract exosomal RNA according to the manufacturer's protocols. Total RNA of cells was extracted using Trizol Reagent (Invitrogen, USA). The quantity and quality of RNA was assessed by NanoDrop® ND-2000 (Thermo Scientific) and Agilent 2100 Bioanalyzer (Agilent Technologies). The Human microRNA Microarray Kit (Agilent Technologies) was used for labeling and hybridization according to the manufacturer's protocol. In brief, 100 ng total RNA was labeled with Cyanine3 (Cy3), re-suspended in hybridization buffer and hybridized to the array platform overnight (20 h) at 55°C in a rotating Agilent hybridization oven using Agilent's recommended hybridization chamber. Subsequently, the microarrays were washed with the Agilent Gene Expression Wash Buffer 1 for 5 min at room temperature. A second washing step was performed with Agilent Gene Expression Wash Buffer 2 warmed to 37°C for 5min. Fluorescence signals after hybridization were detected with a DNA microarray scanner G2505C (Agilent Technologies) using one color scan setting for 8 × 60 K array slides (Scan Area 61 × 21.6 mm, Scan resolution 5 μm, Dye channel is set to Green and Green PMT is set to 100%).

### MiRNA data analysis of A549, A549/DDP cells and their exosomes

The microarray experiment was performed by BGI company (Beijing, China). In order to obtain background subtracted and outlier rejected signal intensities, the scanned microarray images were analyzed and processed with the Agilent feature extraction software (v10.10.1.1) using default parameters (Grid: 046064_D_20121223). The resulting raw Signal intensities (gMedianSignal) were exported to R software and normalized by Quantile normalization method. The pairwise expression fold change and P value were calculated via Student's paired t-test after merging the spots with same Agilent probe ID. Differentially expressed miRNAs were filtered to exclude those less than 1.5-fold changes compared with A549 or exosomes of A549. The microarray data have been submitted to the Gene Expression Omnibus and the data could be obtained by the accession number, GSE85603 and GSE85604.

### Real-time quantitative RT-PCR

Total RNA of cells was extracted using Trizol Reagent (Invitrogen, USA). The Total Exosome RNA and Protein Isolation Kit (Invitrogen, USA) was used to extract exosomal RNA according to the manufacturer's protocols. The expression levels of miRNAs were amplified using SYBR green technique. U6 snRNA was used as endogenous control to normalize miRNA expression in cells, and cel-miR-39 was used to normalize miRNA expression in exosomes between the samples. The primers of the selected miRNAs were synthesized by RiboBio company (Guangzhou, China). Relative miRNA expression was calculated by 2^−ΔΔCt^ method. PCR products were analyzed by agarose gel electrophoresis. All the samples were repeated three times for each miRNA.

### Statistical methods

All the result data were representative of at least three experiments independently and shown as mean ± standard deviation (SD). Statistical analysis was conducted using Student's t-test and one-way ANOVA, and P < 0.05 was considered statistically significant. All of the statistical analyses were performed using SPSS 22.0 software.
